# The systemic impact of periodontitis in about 100,000 patients: associations with heart diseases, cancer, and mortality

**DOI:** 10.1007/s00784-025-06710-w

**Published:** 2025-12-20

**Authors:** Friederike Weinbecker, Susanne Nahles, Robert Preissner, Max Heiland, Florian Kernen, Saskia Preissner

**Affiliations:** 1 Department Oral and Maxillofacial Surgery, Charité – Universitätsmedizin Berlin, Augustenburger Platz 1, 13353 Berlin, Germany; 2 Institute of Physiology and Science-IT, Charité – Universitätsmedizin Berlin, Philippstrasse 12, 10115 Berlin, Germany; 3 Department of Oral and Maxillofacial Surgery, Translational Implantology, Medical Center, Faculty of Medicine, University of Freiburg, Freiburg im Breisgau, Germany

**Keywords:** Real-world evidence, periodontal disease, Neoplasms, Cardiovascular diseases

## Abstract

**Objectives:**

Periodontitis, a chronic inflammatory condition of tooth-supporting tissues, is associated with adverse systemic health outcomes, including cardiovascular disease, malignancies and premature mortality. Large-scale assessments remain limited. We analyzed real-world data of approximately 100,000 matched patients to clarify the broader health impact of periodontitis.

**Study design:**

In this retrospective study, we identified two cohorts − 47,498 patients each with and without periodontitis (ICD-10-CM: K05.6) - from the TriNetX database, matched by age and gender. We evaluated outcomes over 19 years, including heart inflammations, heart disease, head and neck cancer, C-reactive protein (CRP) levels and overall mortality. Propensity score matching, risk analyses and Kaplan–Meier survival statistics were employed.

**Results:**

Compared to controls, patients with periodontitis exhibited higher risks of heart inflammations (OR: 3.06; 95% CI: 2.79–3.35), heart disease (OR: 2.40; 95% CI: 2.33–2.47), overall cancer (OR: 1.64; 95% CI: 1.58–1.71), head and neck cancer (OR: 1.60; 95% CI: 1.31–1.96) and premature mortality (OR: 1.42; 95% CI: 1.36–1.48). Median CRP levels were higher in the periodontitis group (39.30 vs. 35.79; *p* < 0.001).

**Conclusion:**

Periodontitis is associated with significantly increased adverse systemic health outcomes and higher mortality.

**Clinical relevance:**

These findings underscore the importance of preventive oral care and enhanced patient surveillance.

## Introduction

Periodontitis is defined as a chronic multifactorial inflammatory disease associated with a dysbiotic dental biofilm and characterized by a progressive destruction of the tooth-supporting tissue [1]. Its primary features include the loss of gingiva, ligament and alveolar bone, which leads to the typical hallmark of a periodontal pocket [2]. According to the *Global Burden of Disease Study 2017* periodontitis is a widespread condition which affects 50% of the adults over 30 years old worldwide including its mildest forms, meanwhile about 10% to 15% suffer from the severe state of periodontitis [3]. If left untreated, periodontitis can ultimately lead to tooth loss, impacting not only the masticatory function, therefore the nutrition intake and speech impairments but also compromises patients’ self-esteem and overall quality of life [4].

Beyond its local effects, periodontitis has long been linked to various systemic health conditions, sparking interest in the pathogenesis of different diseases, including cardiovascular diseases, heart infections and inflammations and tissue-specific cancer types [1]. In fact, periodontal pathogens such as *Porphyromonas gingivalis* and *Fusobacterium nucleatum* have been found in oral squamous cell carcinoma (OSCC) histological samples as well as in atherosclerotic lesions indicating a possible causative mechanism in the pathogenesis [5]. The periodontal pathogenesis is characterized by an active bacterial subversion of the host immune response, enabling the persistence of pathogens in the local inflammatory environment which can mediate inflammatory complications at local as well as distant sites [6].

Although numerous population-based and practice-based studies have examined associations between periodontitis and systemic diseases, many of these analyses are limited by smaller sample sizes, regional study populations, or heterogeneous definitions of periodontal disease. Large-scale real-world investigations with long-term follow-up and harmonized diagnostic coding remain scarce. By leveraging a multinational federated database with more than 24 million controls and up to 19 years of follow-up, the present study provides a broader and more generalizable assessment of the systemic implications of periodontitis.

In this retrospective study, we utilized the federated TriNetX health research network, which provides access to harmonized electronic medical records from healthcare organizations worldwide. This allowed us to perform a large-scale, real-world outcome comparison based on 47,498 matched patients. The aim of this study was to investigate the associations between periodontitis and a range of systemic health outcomes and to highlight the importance of interdisciplinary collaboration in recognizing and managing the broader health implications of periodontal disease.

## Materials and methods

This retrospective analysis was run on July 18th, 2024, using electronic medical records from the global research platform TriNetX, which includes data from 94 health care organizations (HCOs).

### Cohort definition and database access

Cohorts were defined based on the presence or absence of International Classification of Diseases (ICD-10) code K05.6 (unspecified periodontitis) in conjunction with an inpatient encounter within a 20-year observation period. Because K05.6 does not differentiate between periodontal stages or grades, the case definition may introduce nondifferential misclassification with respect to disease severity, which is an inherent limitation of ICD-coded real-world datasets.

To ensure consistent data availability across all participating institutions, an inpatient encounter was required for both cohorts. Within the TriNetX infrastructure, diagnostic information is frequently stored in encounter-linked format, and inpatient encounters represent the most uniformly coded and complete data elements across HCOs. However, restricting the dataset to individuals with at least one hospitalization may introduce selection toward patients with greater medical needs.

The analysis began at the time of the first occurrence of the index event. For Cohort 1 this included both a diagnosis of unspecified periodontal disease and an inpatient encounter. For Cohort 2, patients had an impatient encounter without a diagnosis of periodontitis. The primary cohort (Cohort 1) included 50,234 patients with a periodontitis diagnosis, while the comparison cohort (Cohort 2) comprised of 23,582,467 patients without a diagnosis of periodontitis. Patients whose index events occurred more than 20 years ago were excluded from the analysis.

To control for potential confounding factors between the two cohorts, propensity score matching was employed. Propensity scores were calculated based on a variety of patient characteristics, including age, gender, diagnosis, and comorbidities such as cardiovascular disease, ensuring comparability between cohorts and enabling more accurate assessment of systemic health outcomes in patients with and without periodontitis. Confounders such as alcohol and nicotine dependence were adjusted. Smoking and alcohol use were identified using ICD-10-CM codes (F17 and Z72.0 for tobacco use; F10 for alcohol-related disorders) and incorporated into the propensity score matching model. This approach reduced baseline imbalance and allowed comparison of outcomes between cohorts with similar demographic and clinical characteristics. After matching each cohort comprised of 47,498 patients. The mean age in both the periodontitis cohort (Cohort 1) and the non-periodontitis cohort (Cohort 2) was 58.30 years (SD +/- 17.10). In both cohorts, 53.10% of participants were female. Outcomes analyzed included heart infections and inflammatory heart conditions, cardiovascular diseases, head and neck cancer, all-cause mortality, and systemic inflammation as indicated by C-reactive protein (CRP) levels. The analysis process included multiple analytical methods, including measures of association, Kaplan-Meier survival analysis, number of instances, and distribution of laboratory results. We calculated the risk difference, risk ratio, and odds ratio of the two cohorts. Additionally, a log-rank test, hazard ratio analysis, and proportionality testing were performed. T-test statistics were used to evaluate differences between cohorts, with outcomes compared using a 95% confidence interval (CI). Because CRP values are typically right-skewed and the TriNetX platform provides only means, standard deviations, and t-tests for laboratory variables, log-transformation or non-parametric modelling could not be performed. Statistical significance was defined as a p-value of less than 0.05. Due to the federated nature of the TriNetX platform, individual patient-level data cannot be exported or accessed externally. Therefore, additional multivariable logistic regression models beyond the built-in analytics cannot be performed. Cox proportional hazards modeling is, however, integrated into the platform and was used for the mortality analyses.

### Ethical approval and consent to participate

This study is declared as non-human subject research due to the de-identification of the data. The study is exempt from informed consent as it involves secondary analysis of existing, de-identified data and does not include any direct interaction with human subjects.

All data were obtained from the TriNetX platform, which complies with the Health Insurance Portability and Accountability Act (HIPAA). The de-identification of patient data follows the standard outlined in Section § 164.514(a) of the HIPAA Privacy Rule and is supported by a formal determination from a qualified expert, as defined in Section § 164.514(b)1. This expert determination was last updated in December 2023.

## Results

**Fig. 1:**
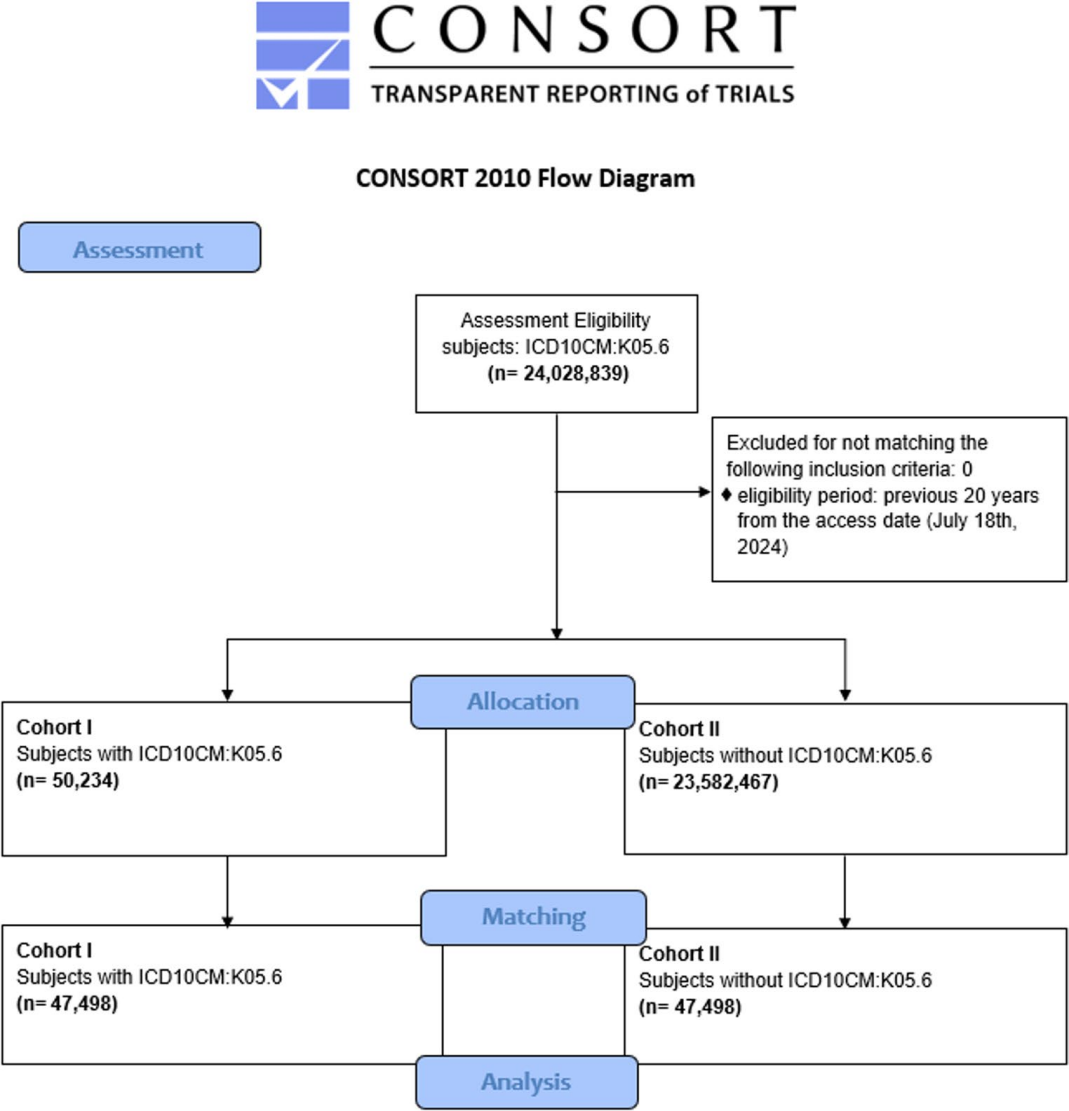
CONSORT flow diagram, major stages indicated in blue boxes

No patients were excluded due to the selected time frame. The initial sample consisted of 50,321 patients with a diagnosis of unspecified periodontal disease (Cohort 1) and 24,028,839 patients without this diagnosis (Cohort 2). After performing propensity score matching, each cohort comprised 47,498 patients, ensuring that age, gender, and other potential confounding factors, such as smoking status and alcohol intake, were statistically comparable between the two groups (Fig. 1). Results are listed in Tables 1 and 2.

### Risk of heart infections/inflammations and heart diseases

Heart infections and inflammations were defined to include diagnoses such as gonococcal heart infection (ICD10CM: A54.83), other specified viral diseases (ICD10CM: B33.8), acute and subacute infective endocarditis (ICD10CM: I33.0), candidal endocarditis (ICD10CM: B37.6), unspecified myocarditis (ICD10CM: I51.4), acute pericarditis (ICD10CM: I30), and chronic adhesive pericarditis (ICD10CM: I31.0). The analysis revealed that the risk of developing these conditions was significantly higher in Cohort 1 (risk = 0.04) compared to Cohort 2 (risk = 0.01). The calculated risk ratio was 2.98 (95% CI 2.723, 3.253), indicating that patients with periodontitis had nearly a threefold increased chance of developing heart infections or inflammations compared to those without periodontitis.

For heart diseases, which were identified based on diagnoses including heart failure (ICD10CM: I50), ill-defined heart disease complications (ICD10CM: I51), other cardiac arrhythmias (ICD10CM: I49), other viral agents as the cause of diseases classified elsewhere (ICD10CM: B97.89), acute myocardial infarction (ICD10CM: I21), angina pectoris (ICD10CM: I20), and unspecified endocarditis (ICD10CM: I38), the results demonstrated that 37.40% of patients in Cohort 1 (risk = 0.37) experienced heart diseases compared with 20.00% in Cohort 2 (risk = 0.20). The risk ratio of 1.87 (95% CI 1.83, 1.91), which translates to an 87.30% higher risk of heart diseases in the periodontitis cohort.

### Risk of cancer and head and neck cancer

The evaluation of cancer risk included all neoplasms (ICD10CM: C00-D49), and head and neck cancer specifically (ICD10CM: C00-C14) were defined as malignant neoplasms of the lip, oral cavity, and pharynx. Patients in Cohort 1 exhibited a significantly higher risk of developing cancer, with a risk of 0.22 compared to 0.14 in Cohort 2. This corresponded to a risk ratio of 1.51 (95% CI 1.5, 1.6), indicating a 50.5% increased risk of cancer in the periodontitis group. In this analysis, 240 patients in Cohort 1 were diagnosed with head and neck cancers, compared to 154 patients in Cohort 2. This analysis yielded a risk ratio of 1.60 (95% CI 1.31, 2), corresponding to a 59.80% higher risk in patients with periodontitis.

### CRP levels

C-reactive protein (CRP) levels, used as a marker for systemic inflammation, were also compared between the cohorts. Patients with periodontitis had a mean CRP level of 39.30 (SD = 62.84), based on 10,039 available outcomes, whereas patients without periodontitis had a mean CRP level of 35.79 (SD = 59.57) from 7,110 outcomes. A t-test comparing the two cohorts resulted in a test statistic of 3.684 with 17,147 degrees of freedom and a p-value of less than 0.001, indicating that the CRP levels were significantly higher in patients with periodontitis. Additionally, inflammation-related outcomes showed a risk ratio of 1.42 (95% CI: 1.38, 1.46), further supporting the association between periodontitis and heightened systemic inflammation.

### Overall and cause-specific mortality

Finally, the mortality analysis demonstrated that patients with unspecified periodontitis had a higher risk of death compared to those without periodontitis. The risk of death in the periodontitis cohort was 0.10, while the risk in the non-periodontitis cohort was 0.08, resulting in a risk ratio of 1.37 (95% CI 1.32–1.43). In addition to the odds ratios, the TriNetX platform provides Cox proportional hazards modeling. The hazard ratio for all-cause mortality was 1.30 (95% CI 1.25–1.36), indicating a significantly elevated mortality hazard among patients with periodontitis. Kaplan–Meier survival analysis further showed lower survival probabilities in the periodontitis group (62.86%) compared to the non-periodontitis group (74.03%), consistent with the hazard ratio estimate.

**Table 1:** Summary of results of death and periodontitis comorbidities

Comorbidity	Cohorts	Events (*n*)	Risk	Risk Difference (95% CI)	Risk Ratio (95% CI)	Odds Ratio (95% CI)
Death	1 w/ periodontitis	4,936	0.10	0.03 (0.03, 0.03)	1.37 (1.32, 1.43)	1.42 (1.36, 1.48)
	2 w/o periodontitis	3,592	0.08			
Heart diseases	1 w/ periodontitis	17,777	0.37	0.17 (0.17, 0.18)	1.87 (1.83, 1.91)	2.40 (2.38, 2.47)
	2 w/o periodontitis	9,490	0.20			
Heart inflammations and infections	1 w/ periodontitis	1,890	0.04	0.03 (0.02, 0.03)	2.98 (2.72, 3.25)	3.06 (2.79, 3.35)
	2 w/o periodontitis	635	0.01			
Cancer	1 w/ periodontitis	5,528	0.22	0.07 (0.07, 0.08)	1.51 (1.46, 1.56)	1.64 (1.58, 1.71)
	2 w/o periodontitis	5,648	0.14			
Head and neck cancer	1 w/ periodontitis	240	0.01	0.00 (0.00, 0.00)	1.60 (1.31, 1.96)	1.60 (1.31, 1.96)
	2 w/o periodontitis	154	0.00			
Inflammation	1 w/ periodontitis	10,653	0.22	0.07 (0.06, 0.07)	1.42 (1.38, 1.46)	1.54 (1.49, 1.60)
	2 w/o periodontitis	7,500	0.16			

**Table 2:** CRP levels

Comorbidity	Cohorts	Events (*n*)	Mean	Standard Deviation
CRP	1 w/ periodontitis	10,039	39.30	62.84
	2 w/o periodontitis	7,110	35.79	59.57

## Discussion

In this large retrospective cohort spanning 6,935 days (approximately 19 years), individuals with periodontitis exhibited a significantly elevated risk of cardiovascular diseases, cancer and premature mortality compared to those without the condition. These findings underscore the potential systemic impact of periodontitis and align with growing evidence that chronic oral inflammation contributes to adverse health outcomes [7–9]. Although the absolute difference in mortality between groups was modest (10% vs. 8%), the effect remained statistically significant and consistent across analytical approaches, including the Cox proportional hazards model.

Several biological mechanisms may underlie these associations.

Periodontitis is characterized by a shift toward microbial dysbiosis in the subgingival biofilm, which provokes persistent local inflammation and impairs host immune regulation [10, 11].

Keystone pathogens such as *Porphyromonas gingivalis* and *Fusobacterium nucleatum* can invade epithelial cells and induce (epi)genetic alterations, leading to endothelial dysfunction and atherosclerotic plaque formation [12]. Along with these bacteria, metabolic by-products and endotoxins can induce mutations in proto-oncogenes and tumor suppressor genes or interfere with molecular pathways involved in cell proliferation and survival [13–15]. Indeed, a recent meta-analysis reported that periodontitis is an independent risk factor for head and neck squamous cell carcinoma, with a pooled OR of 3.17 (95% CI 1.78–5.64) [16].

Furthermore, these pathogens and their virulence factors may disseminate via hematogenous and enteral routes, potentially promoting inflammatory cascades at distant organ sites [17]. For example, *Porphyromonas gingivalis* and *Fusobacterium nucleatum* have been isolated from inflammatory bowel diseases and detected in premalignant lesions like colorectal adenomas and colorectal cancers [18]. Consistent with these observations, periodontitis has been associated with local neoplastic alterations and may also show associations with neoplastic processes at distant sites [19, 20]. The elevated CRP levels observed in the periodontitis cohort support the biological plausibility of this association, as chronic systemic inflammation represents a well-established pathway linking periodontal disease with cardiovascular and neoplastic outcomes.

Despite the robust size and observation period of the cohort, several limitations should be considered when interpreting our findings. First, the requirement of an inpatient encounter for cohort inclusion may introduce selection bias, as it restricts the study population to individuals with at least one hospitalization. This requirement may introduce collider bias by selecting for patients with greater medical needs and reduces the generalizability of our findings to healthier, community-based populations.

Second, reliance on ICD-10 codes from the real-world database TriNetX may introduce misclassification bias. The available data did not allow for classification according to the current staging and grading system [21], nor for assessing disease severity in detail, which limits the interpretation of our findings, particularly given the fact that periodontitis is a highly heterogeneous disease with a broad spectrum of clinical presentations and progression patterns. Third, the retrospective observational design limits causal inference. Although we observed strong associations between periodontitis and systemic outcomes, residual confounding by unmeasured factors (e.g. socioeconomic status, access to care, comorbidities) cannot be excluded [22]. Finally, because age itself is a major risk factor for periodontitis [23, 24], incident cases developing during follow-up may have been misclassified at baseline, potentially diluting true associations.

Our findings corroborate previous studies that link periodontal inflammation to systemic disease, yet they also highlight the need for standardized and comprehensive periodontal assessment in epidemiological research [25]. Future investigations should incorporate the 2017 World Workshop on the Classification of Periodontal and Peri-Implant Diseases and Conditions to ensure consistency and comparability across cohorts and should include objective measures such as clinical attachment loss, probing depth, and circulating C-reactive protein levels to better characterize disease severity and systemic inflammatory burden [26, 27]. Interventional studies are needed to investigate underlying causal mechanisms. A limited number of randomized controlled trials have demonstrated reductions in surrogate markers of cardiovascular risk following intensive periodontal therapy [28–30], however, large-scale and long-term trials assessing hard clinical endpoints remain lacking.

From a clinical and public health perspective, these results reinforce the importance of early detection and management of periodontitis. Although concerns remain regarding the socioeconomic costs of widespread screening and potential overtreatment [31], targeted periodontal evaluation during routine dental visits may facilitate timely interventions that could mitigate long-term systemic risks. Cost-effectiveness analyses comparing various screening modalities and treatment thresholds are urgently needed to inform policy and optimize resource allocation [32]. 

## Conclusions

The study underlines the significant systemic impact periodontitis has on associated health outcomes. Patients with periodontitis were found to have a notably higher chance of developing heart infections, cardiovascular diseases, head and neck cancers and overall mortality over nearly two decades of follow-up. The findings underscore the importance of early detection and increased surveillance of periodontitis to prevent broader health risks for patients. Calling for future research to fully elucidate the biological mechanisms and to explore a more detailed assessment of the progression and severity of periodontitis.

## Data Availability

The dataset presented in this study can be retrieved upon reasonable request.

## References

[1] Romandini M, Baima G, Antonoglou G, Bueno J, Figuero E, Sanz M (2021) Periodontitis, Edentulism, and risk of mortality: A systematic review with Meta-analyses. J Dent Res 100(1):37–49 32866427 10.1177/0022034520952401

[2] Kinane DF, Stathopoulou PG, Papapanou PN (2017) Periodontal diseases. Nat Reviews Disease Primers 3(1):17038 28805207 10.1038/nrdp.2017.38

[3] Collaborators GBDOD, Bernabe E, Marcenes W, Hernandez CR, Bailey J, Abreu LG et al (2020) Global, Regional, and National levels and trends in burden of oral conditions from 1990 to 2017: A systematic analysis for the global burden of disease 2017 study. J Dent Res 99(4):362–373 32122215 10.1177/0022034520908533 PMC7088322

[4] Farran M, Neppelberg E, Loes S, Aarstad AKH, Moe SE, Aarstad HJ (2024) Periodontitis and dental quality of life predict long-term survival in head and neck cancer. BMC Oral Health 24(1):1406 39563313 10.1186/s12903-024-05170-0 PMC11575175

[5] Han YW, Wang X (2013) Mobile microbiome: oral bacteria in extra-oral infections and inflammation. J Dent Res 92(6):485–491 23625375 10.1177/0022034513487559 PMC3654760

[6] Hajishengallis G (2015) Periodontitis: from microbial immune subversion to systemic inflammation. Nat Rev Immunol 15(1):30–44 25534621 10.1038/nri3785 PMC4276050

[7] Gao S, Tian J, Li Y, Liu T, Li R, Yang L et al (2021) Periodontitis and number of teeth in the risk of coronary heart disease: an updated Meta-Analysis. Med Sci Monit 27:e930112 34421117 10.12659/MSM.930112 PMC8394608

[8] Kalhan AC, Wong ML, Allen F, Gao X (2022) Periodontal disease and systemic health: an update for medical practitioners. Ann Acad Med Singapore 51(9):567–574 36189701

[9] Michaud DS, Fu Z, Shi J, Chung M (2017) Periodontal Disease, tooth Loss, and cancer risk. Epidemiol Rev 39(1):49–58 28449041 10.1093/epirev/mxx006 PMC5868279

[10] Kwon T, Lamster IB, Levin L (2021) Current concepts in the management of periodontitis. Int Dent J 71(6):462–476 34839889 10.1111/idj.12630 PMC9275292

[11] Lamont RJ, Koo H, Hajishengallis G (2018) The oral microbiota: dynamic communities and host interactions. Nat Rev Microbiol 16(12):745–759 30301974 10.1038/s41579-018-0089-x PMC6278837

[12] Baima G, Minoli M, Michaud DS, Aimetti M, Sanz M, Loos BG et al (2024) Periodontitis and risk of cancer: mechanistic evidence. Periodontol 2000 96(1):83–94 38102837 10.1111/prd.12540 PMC11579815

[13] Colonia-García A, Gutiérrez-Vélez M, Duque-Duque A, De Andrade CR (2020) Possible association of periodontal disease with oral cancer and oral potentially malignant disorders: a systematic review. Acta Odontol Scand 78(7):553–559 32552160 10.1080/00016357.2020.1774076

[14] Ha NH, Woo BH, Kim DJ, Ha ES, Choi JI, Kim SJ et al (2015) Prolonged and repetitive exposure to Porphyromonas gingivalis increases aggressiveness of oral cancer cells by promoting acquisition of cancer stem cell properties. Tumor Biology 36(12):9947–9960 26178482 10.1007/s13277-015-3764-9

[15] Anil S, Preethanath VS, Farraj Aldosari RSSP (2012) AA. The emerging concepts on the impact of periodontitis on systemic health. InTech

[16] Gopinath D, Kunnath Menon R, Veettil K, George Botelho S, Johnson M (2020) Periodontal diseases as putative risk factors for head and neck cancer: systematic review and Meta-Analysis. Cancers 12(7):1893 32674369 10.3390/cancers12071893 PMC7409086

[17] Nwizu N, Wactawski-Wende J, Genco RJ (2020) Periodontal disease and cancer: epidemiologic studies and possible mechanisms. Periodontol 2000 83(1):213–233 32385885 10.1111/prd.12329 PMC7328760

[18] Kostic AD, Chun E, Robertson L, Glickman JN, Gallini CA, Michaud M et al (2013) Fusobacterium nucleatum potentiates intestinal tumorigenesis and modulates the tumor-immune microenvironment. Cell Host Microbe 14(2):207–215 23954159 10.1016/j.chom.2013.07.007 PMC3772512

[19] Hajishengallis G, Chavakis T (2021) Local and systemic mechanisms linking periodontal disease and inflammatory comorbidities. Nat Rev Immunol 21(7):426–440 33510490 10.1038/s41577-020-00488-6 PMC7841384

[20] Michaud DS, Kelsey KT, Papathanasiou E, Genco CA, Giovannucci E (2016) Periodontal disease and risk of all cancers among male never smokers: an updated analysis of the health professionals Follow-up study. Ann Oncol 27(5):941–947 26811350 10.1093/annonc/mdw028 PMC4843185

[21] Tonetti MS, Greenwell H, Kornman KS (2018) Staging and grading of periodontitis: framework and proposal of a new classification and case definition. J Periodontol 89(Suppl 1):S159–S72 29926952 10.1002/JPER.18-0006

[22] Ludwig RJ, Anson M, Zirpel H, Thaci D, Olbrich H, Bieber K et al (2025) A comprehensive review of methodologies and application to use the real-world data and analytics platform TriNetX. Front Pharmacol 16:1–16 10.3389/fphar.2025.1516126 PMC11931024 40129946

[23] Nazir M, Al-Ansari A, Al-Khalifa K, Alhareky M, Gaffar B, Almas K (2020) Global prevalence of periodontal disease and lack of its surveillance. Sci World J 2020:1–8 10.1155/2020/2146160 PMC7275199 32549797

[24] Tadjoedin FM, Fitri AH, Kuswandani SO, Sulijaya B, Soeroso Y (2017) The correlation between age and periodontal diseases. J Int Dent Med Res 10(2):327

[25] Savage A, Eaton KA, Moles DR, Needleman I (2009) A systematic review of definitions of periodontitis and methods that have been used to identify this disease. J Clin Periodontol 36(6):458–467 19508246 10.1111/j.1600-051X.2009.01408.x

[26] Caton JG, Armitage G, Berglundh T, Chapple ILC, Jepsen S, Kornman KS et al (2018) A new classification scheme for periodontal and peri-implant diseases and conditions – Introduction and key changes from the 1999 classification. J Clin Periodontol 45(S20):S1–S8 29926489 10.1111/jcpe.12935

[27] Bárcena García M, Cobo Plana JM, Rodríguez Cagiao G, Arcos González PI (2024) Epidemiological methods used in the periodontal health research in military personnel: a systematic review. BMJ Military Health 170(1):72–77 34921095 10.1136/bmjmilitary-2021-001977 PMC10850676

[28] Corlin L, Ruan M, Tsilidis KK, Bouras E, Yu YH, Stolzenberg-Solomon R et al (2021) Two-Sample Mendelian randomization analysis of associations between periodontal disease and risk of cancer. JNCI Cancer Spectr 5(3):1–8 10.1093/jncics/pkab037 PMC8242136 34222791

[29] Santos-Paul MA, Neves RS, Gowdak LHW, De Paula FJ, David‐Neto E, Bortolotto LA et al (2019) Cardiovascular risk reduction with periodontal treatment in patients on the waiting list for renal transplantation. Clin Transpl 33(8):1–11 10.1111/ctr.13658 31271675

[30] Hada DS, Garg S, Ramteke GB, Ratre MS (2015) Effect of Non-Surgical periodontal treatment on clinical and biochemical risk markers of cardiovascular disease: A randomized trial. J Periodontol 86(11):1201–1211 26205747 10.1902/jop.2015.150249

[31] Braegger U (2005) Cost–benefit, cost-effectiveness and cost–utility analyses of periodontitis prevention. J Clin Periodontol 32(s6):301–313 16128845 10.1111/j.1600-051X.2005.00802.x

[32] Tonetti MS, Jepsen S, Jin L, Otomo-Corgel J (2017) Impact of the global burden of periodontal diseases on health, nutrition and wellbeing of mankind: A call for global action. J Clin Periodontol 44(5):456–462 28419559 10.1111/jcpe.12732

